# Agricultural chemical use and the rural-urban divide in Canada

**DOI:** 10.1080/21645698.2024.2318876

**Published:** 2024-02-20

**Authors:** Stuart J. Smyth, Sylvain Charlebois

**Affiliations:** a Department of Agricultural and Resource Economics, University of Saskatchewan, Saskatoon, Saskatchewan, Canada; b Faculty of Management, Dalhousie University, Hlaifax, Nova Scotia, Canada

**Keywords:** Crop inputs, disinformation, environmental non-governmental organizations, food production, innovation, precautionary-based, science-based, sustainability

## Abstract

Innovation is of fundamental importance for improving food production, as well as sustainability food production. Since 1960, food production has benefited from innovations in plant breeding technologies, fertilizer, chemicals and equipment. These innovations have dramatically increased food production, while the amount of land used has minimally increased. However, future food production increases are jeopardized from widening knowledge gaps between rural food producers and large urban food consuming populations. Over time, that gap has fueled disinformation. The development of disinformation business models contributes to urban consumers receiving inaccurate information about the importance of inputs essential to food production, resulting in political pressures being applied that are targeted at reductions in the use of many food production inputs. The use of chemicals are a frequent target of disinformation campaigns. This article examines how the lack of government clarity about the safe use of chemicals contributes to a lack of public information.

## Introduction

Food production, food security and food safety are three crucial aspects of the global agriculture and agri-food industries. So important are these three aspects, that they anchor many of the Sustainable Development Goals put forth by the United Nations in 2015, especially the second and third goals, which are respectively reducing hunger and improving human health.^a^^a^See: https://sdgs.un.org/goals. Globally, significant increases in food production have occurred since 1960, when food production became decoupled from increased land to produce food. Between 1960 and 2020, food production increased by 390%, while only 10% more land is used to produce food.^1^ The dramatic increase in food production is the result of numerous innovative advancements, most notably in the technologies used to develop new crops, more efficient weed control from chemicals and improved crop nutrients from fertilizers.^2,3^

In the last six decades, there has been a notable increase in food production; however, a persistent issue remains with over 800 million individuals experiencing food insecurity in 2022.^4^ Several factors have contributed to this situation, including disruptions in supply chains during the COVID-19 pandemic and the consequences of Russia’s invasion of Ukraine, leading to an estimated 828 million people facing food insecurity. Disturbingly, the number of food-insecure individuals has shown an upward trend since 2015. Additionally, an alarming trend has emerged within the policies and regulations implemented by many governments, as they deviate from empirically-based approaches.

Of particular concern is the domain of agriculture and food crop production, where there is a noteworthy instance in the European Union’s Farm to Fork Strategy (F2F Strategy). This strategy advocates for reductions in the use of chemicals, decreased chemical toxicity, lower fertilizer utilization and a shift toward increased organic production. Regrettably, these propositions lack adequate reference to empirically-based evidence supporting the effectiveness of the F2F Strategy.^5^ Furthermore, this strategy exempts all organic chemicals, which, contrary to synthetic alternatives, are deemed to be more highly toxic and have been identified as carcinogenic.^6^ The growing tendency to veer away from empirically-based policy development raises concerns about potential ramifications. Such a shift may result in increased market efficiencies, but it also carries the risk of unforeseen consequences. For instance, the implementation of policies that lack a solid empirical foundation may lead to unintended effects, including detrimental impacts on human health due to heightened exposure to carcinogenic organic chemicals. Therefore, it is crucial for policymakers to reconsider their approach and place greater emphasis on evidence-based decision-making in order to address the complex challenges surrounding food security and safeguard public welfare.

The transition away from empirically-based agricultural policy to precautionary-based policy has been shown to have damaging production impacts. Empirical-based policies, regulations and agreements underpinned the development of the 20^th^ century, with the establishment of organizations like the World Trade Organization, the International Treaty on Plant Genetic Resources for Food and Agriculture and the Organization for Economic Cooperation and Development. Through the establishment of empirically-based rules and the codification of regulations and international commodity trade, efficiencies were gained by all participatory countries. However, the significant successes of these 20^th^ century institutions created the luxury of societies to no longer recall the challenges that existed prior to the establishment of empirically-based institutions, allowing them to propose precautionary-based mechanisms as an alternative means of enacting policies and regulations.

In numerous countries, the utilization of agricultural chemicals has come under considerable scrutiny, with mounting pressures for reductions and even outright bans. This shift is primarily attributed to disinformation campaigns orchestrated by environmental non-governmental organizations (ENGOs).^7^ These campaigns have had the effect of misleading various stakeholders, including voters, politicians and policymakers, leading to the formulation of agricultural policies that lack a solid grounding in empirical evidence. This raises pertinent questions regarding the underlying drivers behind this transition away from evidence-based approaches as the foundation for sound policies and regulations. Moreover, it is essential to comprehend the potential implications of a future characterized by the prevalence of disinformation, precautionary policy and an increased regulatory burden.

This article endeavors to delve into the dynamics of the relationship between two conflicting perspectives within this realm: on one hand, the empirically-based safe use of agricultural chemicals, as advocated by Canadian farmers, and on the other hand, the precautionary-based advocacy of ENGOs. By exploring this context, the aim is to shed light on the factors influencing the prevalent narrative and its consequences for agricultural practices and regulations. It is crucial to critically examine the motivations driving the dissemination of misinformation and to assess the implications of adopting a precautionary stance without a robust evidentiary foundation. The implications of such developments could significantly impact agricultural systems, farming communities and the broader environmental landscape, thereby warranting a comprehensive investigation of this intricate issue.

## Plant Breading

Nature is relentless in how crop yields are consistently threatened from combinations of weeds, insects and disease. Weeds rapidly germinate in the spring and left uncontrolled, can out-compete domesticated crops for moisture and nutrients. Studies based on the impacts of uncontrolled weeds in Africa agriculture have quantified yield reductions of up to 80% in the worst instances.^8,9,10,11^ Depending on the crop, yield loss from weeds can be as high as 40%, but more typically range from 3–25%.^12^ Diseases have been reported to have even higher impacts than weeds in terms of yield reductions, with losses of 40–60% reported in soybeans.^13^ Insect controls are also an important aspect of good agronomic practices, as left uncontrolled yields are further reduced. This means that without the proper care and attention to the health of a crop, yields can quickly decline. The range of losses is summed up in the Table 1. It would be highly unlikely that the maximum impact from weeds, insects and disease would occur within the same growing season, but as climates change, there is a greater potential for the impacts from these crop stresses to increase.

**Table 1.** Factors affecting crop loss.

Crop	Weeds	Insects	Plant Disease	Cumulative Loss
Vegetables	8–13%^1^	4–21%^1^	8–23%^1^	20–57%
Soybeans	10–37%^2^	0–11%^3^	40–60%^4^	50–100%
Corn	50%^5^	15–50%^4,6^	8–14%^7^	73–100%
Wheat	5–20%^8^	5–20%^9^	0–16%^10^	10–56%
Canola	40%^11^	10–50%^12^	18–99%^13^	68–100%
Rice	37–50%^14^	28%^15^	15–60%^16^	80–100%
Range	5–50%	0–50%	0–99%	10–100%

1. Howard et al.^14^ 2. Soltani et al.^12^ 3. Musser et al.^15^ 4. Bradley et al.^13^ 5. Soltani, et al.^16^ 6. Morgan^17^ 7. Mueller et al.^18^ 8. Flessner et al.^19^ 9. Deutsch et al.^20^ 10. Ficke et al.^21^ 11. Harker ^22^ 12. Sekulic and Rempel ^23^ 13. Wang et al.^24^ 14. Zoschke ^25^ 15. Mondal ^26^ 16. Baite et al.^27^

Weeds typically germinate earlier than planted crops, grow at faster rates than seeded crops and produce more seeds, therefore proper weed control is necessary for farmers. Weeds like palmer amaranth are a particularly noxious weed in the southern United States, which can produce up to 250,000 seeds per plant per summer.^28^ Kochia, a common weed in Western Canada is capable of producing 25,000 seeds a summer.^29^ The lack of effective weed control in the instance of weeds such as this, can result in areas of land that are no longer capable of producing crops due to the high presence of weeds. A good yielding variety of wheat typically produces 25–30 kernels of wheat per plant per season, with an exceptional yield of 40 kernels. When weeds annually produce thousands of times more seeds than a crop, the lack of efficient weed control practices can quickly reduce yields and revenues for farmers.

The commercialization of genetically modified (GM) crops has made significant contributions to reducing the use of chemicals as it relates to food production, particularly the use of insecticides. An assessment of 147 publications on changes in chemical use following the commercialization of GM crops, quantified an overall reduction in the use of chemicals by 37%.^30^ Research from Western Canada, undertaken a decade after the commercialization of GM canola, identified similar results. Comparing herbicide use in 1995 with 2005, identified that with canola acres being similar in size, chemical use had declined by 1.3 million kg of chemical active ingredient.^31^ This represents a decrease in chemical use of 38% between the two periods. Further to this, the environmental impact from the chemicals applied declined by 53%,^32^ providing benefits to farm workers, the environment and consumers.

A robust assessment of the global impacts of banning glyphosate are estimated to result in a farm income loss of US$6.7 billion annually.^33^ Subsequently, such a ban would have damaging impacts on the production of food, through reduced yields of soybean (18.6 million tonnes), corn (3.1 million tonnes) and canola (1.4 million tonnes). The cost of not adopting chemical reducing GM crops is difficult to accurately assess, as the lack of a counterfactual is not available. One study that has examined the costs of not adopting beneficial innovations examined the economic and environmental costs of Australia’s moratorium on GM canola adoption. Australian regulators approved the commercial production of GM canola in 2004, however the Australian canola industry believed there would be premiums for non-GM canola oil in Asia, so a moratorium was implemented on the production of GM canola in Australia. The moratorium lasted for several years in key canola producing states of Victoria, New South Wales and Western Australia, but was not completely removed until 2021 in South Australia.

Biden et al.^34^ established the GM canola adoption rate in Western Canada over its first decade of 1997–2007 and compared this rate with the 2004–2014 Australian adoption rate, allowing them to estimate the economic and environmental costs of the moratorium. After a decade of full and partial state moratorium, the estimated costs include: the application of an additional 6.5 million kg of chemicals; 7 million additional field passes were made, requiring 8.7 million liters of diesel; 24 million kg of greenhouse gases were released; the environmental impact of the additional chemicals applied was 14% higher; and Australian farmers lost the opportunity to increase their farm revenues by A$485 million. This estimate of the costs of not adopting innovative technologies such as GM crops, highlights the significant increase in chemical use and environmental impacts from ignoring evidence.

The advancement of crop production technologies and inputs has undoubtedly played a pivotal role in augmenting overall crop yields. However, a concerning trend has emerged in certain governments’ policy approaches, wherein a shift away from empirical evidence that underpins these productivity gains is observed. Instead, a precautionary-based stance has been adopted, as exemplified by Sri Lanka’s ban on synthetic chemicals and fertilizers, which led to a substantial 54% reduction in crop production in 2022.^35^ Consequently, after one year, Sri Lanka was compelled to reverse this policy due to the severe repercussions it inflicted on food production.

Similarly, the French government imposed a ban on neonicotinoids, a category of pesticides, in 2014. With a lack of viable insecticide alternatives, sugar beet production steadily declined following the phased-in ban, resulting in a remarkable 50% reduction in sugar beet output by 2020, merely three years after the ban’s full implementation.^36^ Consequently, the French government had to rescind the earlier ban and authorize the use of neonicotinoids for a subsequent three-year period to mitigate the adverse consequences on sugar beet production. With the lack of access to neonicotinoid chemicals, farmers were forced to rely on older, more environmental toxic and less efficient chemicals.

The transition by certain governments from empirically-based policies to precautionary-based policies has exhibited profound detrimental effects on both food production and the environment. Notably, ENGOs have played a significant role in this shift, employing deliberate disinformation campaigns to influence governments, consumers, and policymakers. Consequently, policies have been formulated that not only curtail food production but also exacerbate the environmental impacts associated with food production processes.

The ramifications of such policy shifts demand meticulous attention and scrutiny, as they have far-reaching implications for food security, environmental sustainability and overall agricultural productivity. Policymakers and stakeholders alike must be vigilant in assessing the validity and robustness of evidence behind proposed policy measures to ensure the formulation of effective and sustainable strategies that promote both food security and environmental conservation.

## Policy Analysis and Implications

In Canada, the conflict between Environmental and Climate Change Canada (ECCC) and the Pest Management Regulatory Agency (PMRA) concerning the use of chemicals on crown land exemplifies the complexities surrounding food production policies. While the PMRA, as the regulatory authority, approves chemicals for safe use, the ECCC seeks to exert greater influence in this domain. The proposal to prohibit chemicals for “cosmetic” purposes on crown land despite their safety approvals sends a conflicting message to the agricultural community and the public.^37^

Strategies to navigate current challenges and achieve a balance in Canadian food production policies are required. For starters, an enhanced science communication approach would be necessary. To bridge the knowledge gap between rural food producers and urban consumers, there is a need for effective science communication strategies.^38^ Government agencies, academia, research institutions and agricultural organizations should collaborate to communicate the importance of various agricultural inputs, such as chemicals, in a transparent and accessible manner. Emphasizing the scientific evidence supporting their safe use can help dispel misinformation and foster informed decision-making.

Health Canada has undertaken two extensive reviews on the impacts of glyphosate use in Canada. In 2017, Health Canada completed a risk assessment, concluding that glyphosate did not provide a risk to human health or the environment and approved glyphosate for a further 15 years of use.^39^ This ruling was challenged by numerous ENGOs based in Quebec, resulting in the PMRA conducting a second risk assessment in 2019, concluding exactly the same outcome as the 2017 assessment. In the 2019 risk assessment announcement, the PMRA strongly states: “[n]o pesticide regulatory authority in the world currently considers glyphosate to be a cancer risk to humans at the levels at which humans are currently exposed.”^40^ The cost of the second risk assessment was not insignificant, however, it confirmed the robustness of the science-based assessment methodology. Regrettably, Canadian ENGOs fail to acknowledge this conclusive evidence-based statement and communicate disinformation about glyphosate use in Canada.

To support a different communications approach, the conceptualization of sustainability in the context of Canadian agriculture necessitates a paradigm shift. The notion of sustainability comprises three fundamental dimensions: society, economy and environmental stewardship. Therefore, for agriculture to genuinely embody sustainability, it must effectively address these three interrelated facets.^41^

Numerous studies have highlighted that agriculture demonstrates a notable capacity to uplift the income levels of impoverished individuals, surpassing other economic sectors in this regard. But this would not suggest that farmers or agribusinesses are the enemy.

Truly sustainable agriculture necessitates the harmonious integration of social, environmental and economic interests. Its overarching objectives encompass ensuring sufficient food provision for all members of society, alleviating communities from the clutches of poverty, enhancing the quality of life for farming families and employing agricultural practices that promote soil health while concurrently reducing dependency on fossil fuels to secure environmental sustainability. This may need to underscore policy development more often.

Public education and outreach should also be a priority. Implementing targeted public education campaigns on agricultural practices and innovations can help create awareness and understanding among consumers.^42,43^ Engaging the public in dialogue about the benefits and risks associated with different agricultural inputs will foster trust and confidence in the agricultural sector. Facilitating meaningful dialogue and collaboration between policymakers, farmers, industry stakeholders and environmental groups can lead to more balanced and pragmatic policies. Involving all relevant parties in the decision-making process will ensure that regulations consider both environmental concerns and the practical realities of agricultural production.

The regulatory approval process for agricultural inputs should be science-based and transparent. Regulatory agencies, such as the PMRA, should continue to conduct thorough risk assessments based on the latest scientific research to ensure the safety and effectiveness of chemicals used in agriculture.^44^

Coupled with these pillars, continued investment in agricultural research and innovation is crucial for finding alternative solutions to reduce chemical dependency while increasing productivity. Research on new plant varieties, precision agriculture technologies and sustainable farming practices can drive agricultural growth and sustainability.

It is essential to consider the global competitiveness of Canadian crop and food production in the context of regulatory burdens. As Canada aims to maintain its position as a competitive player in the global agricultural market, policymakers must strike a balance between environmental concerns and practical chemical use.

Excessive regulatory burdens can potentially hinder innovation and productivity in the agricultural sector. Therefore, while environmental considerations are crucial, it is essential to adopt a risk-based approach that weighs the benefits of agricultural inputs against their potential risks.^45^ By utilizing the latest scientific evidence and conducting rigorous risk assessments, policymakers can develop regulations that maintain environmental standards while supporting the productivity and competitiveness of Canadian farmers.

Navigating the challenges posed by the knowledge gap, disinformation campaigns and environmental concerns requires a multifaceted approach. By prioritizing science-based decision-making, fostering public education and collaboration and incentivizing sustainable practices, Canada can strike a balance between environmental protection and practical chemical use in food production.

As policymakers design regulations and policies, it is crucial to keep in mind the global competitiveness of Canadian agriculture. Striking the right balance will ensure that Canadian farmers can continue to thrive in a competitive international market while upholding their commitment to sustainable and responsible agricultural practices. By embracing innovation, informed policymaking and collaborative efforts, Canada can chart a path toward a resilient and globally competitive agricultural future.

## Conclusion

The disinformation-driven wedge between urban consumers and rural food producers poses significant challenges to evidence-based policymaking. As the demand for sustainable and safe food production practices grows, it is crucial to address this knowledge gap and foster a better understanding of the importance of various agricultural inputs, including chemicals, in achieving these goals. To address this issue, policymakers must prioritize science-based decision-making over precautionary-based approaches. Emphasizing the use of credible scientific evidence and expert analysis will help counteract the influence of disinformation campaigns and prevent misguided policy decisions that may impede agricultural progress.

Innovation remains vital to the future of food production, ensuring sustainability and meeting the needs of an ever-expanding global population. However, the knowledge gap between rural food producers and urban consumers, coupled with the prevalence of disinformation campaigns, poses significant threats to evidence-based policymaking in the agricultural sector. By recognizing the implications of disinformation and the conflicting messages sent by different regulatory bodies, policymakers can take strides toward implementing robust and science-based policies, thereby securing a sustainable and prosperous future for Canadian food production.
